# A vaccine with Aβ oligomer-specific mimotope attenuates cognitive deficits and brain pathologies in transgenic mice with Alzheimer’s disease

**DOI:** 10.1186/s13195-017-0267-5

**Published:** 2017-06-07

**Authors:** Shao-wei Wang, Dong-qun Liu, Ling-xiao Zhang, Mei Ji, Yang-xin Zhang, Quan-xiu Dong, Shu-ying Liu, Xi-xiu Xie, Rui-tian Liu

**Affiliations:** 1 National Key Laboratory of Biochemical Engineering, Institute of Process Engineering, Chinese Academy of Sciences, Haidian District, Beijing, 100190 China; 2 School of Life Science, Anhui Agricultural University, Hefei, 230036 China; 3 School of Life Science, Ningxia University, Yinchuan, 750021 China

**Keywords:** Alzheimer’s disease, β-amyloid oligomer, Mimotope, *Saccharomyces cerevisiae*, Vaccine

## Abstract

**Background:**

β-Amyloid peptide (Aβ) oligomers are initial factors used to induce Alzheimer’s disease (AD) development, and Aβ monomers have normal physiological function. The antibodies or vaccines against Aβ monomers have serious problems, such as side effects and low curative effects. Therefore, it is essential to specifically target Aβ oligomers rather than monomers for the treatment of AD.

**Methods:**

The mimotopes of Aβ oligomers were obtained by panning the phage-displayed random peptide libraries using oligomer-specific antibodies as targets and expressed on the surface of EBY100 *Saccharomyces cerevisiae* to generate yeast cell base vaccines. One vaccine (AOE1) induced antibodies specifically against Aβ oligomers and was selected for further study. The APP/PS1 mice were subcutaneously immunized with AOE1 eight times. The levels and characteristics of antibodies induced by AOE1 were determined by enzyme-linked immunosorbent assay. The effect of AOE1 on the cognitive deficits of AD mice was tested by novel object recognition (NOR) and Y-maze. Dot blot analysis, Western blot analysis, and immunohistochemistry were applied to measure the effects of AOE1 on Aβ pathologies, neuroinflammation, and microhemorrhages in the brains of AD mice.

**Results:**

Eight mimotope candidates of Aβ oligomers were selected and expressed on EBY100 *S. cerevisiae*. Only AOE1 vaccine containing mimotope L2 induced antibodies that specifically recognized Aβ42 oligomers rather than monomers. AOE1 immunization significantly increased the AD mice’s exploration times for the novel object in the NOR test and the choices for new arms in the Y-maze test, and it reduced levels of Aβ oligomers and glial activation in the AD mouse brains. No activation of Aβ-specific T cells and microhemorrhages was observed in their brains following AOE1 vaccination.

**Conclusions:**

AOE1 is the first vaccine applying the oligomer-specific mimotope as an immunogen, which could induce antibodies with high specificity to Aβ oligomers. AOE1 immunization attenuated Aβ pathologies and cognitive deficits in AD mice, decreased the overactivation of glial cells, and did not induce microhemorrhage in the brains of AD mice. These findings suggest that AOE1 may be a safer and more effective vaccine for AD treatment.

## Background

Alzheimer’s disease (AD) is the most prevalent dementia that seriously threatens the health and life of the elderly [1]. The hallmark pathologies of AD are neuronal extracellular senile plaques consisting of β-amyloid peptide (Aβ) aggregates and intracellular neurofibrillary tangles consisting of abnormally hyperphosphorylated tau protein [2]. Aβ oligomers, aggregated from Aβ monomers, are considered to be the initial cause of AD by inducing tau hyperphosphorylation, oxidative stress, inflammatory response, synaptic dysfunction, and subsequent neurodegeneration that underlie the progression of AD [3, 4]. Aβ is a proteolytic fragment of the amyloid precursor protein (APP) by the sequential enzymatic actions of β-secretase and γ-secretase [5]. APP and Aβ play trophic roles in the development of neurons and synapses [6, 7]. Aβ may exist in several forms, including monomers, oligomers, and fibrils, whereas only the oligomeric forms were considered to be more neurotoxic [8].

Anti-Aβ immunotherapy is an efficient way to clear the Aβ burden and has promising applications in AD treatment. However, the risk of autoimmunity and notable side effects, as well as uncertain therapeutic effects, have restricted the development of immunotherapy against Aβ [9]. The first Aβ vaccine, AN1792 using Aβ_42_ fibrils as an immunogen, significantly reduced the amyloid burden in AD transgenic mice after vaccination [10]. Unfortunately, AN1792 was terminated in clinical trials because of meningoencephalitis that occurred in 6% of immunized patients with AD [11]. Subsequent research indicated that T-cell-mediated autoimmunity induced by the self-antigen Aβ_1–42_ was the main cause of this serious adverse effect [12]. To avoid T-cell autoimmunity, the second generation of Aβ vaccines was developed by conjugating a B-cell epitope of Aβ_42_ with a carrier [13]. However, the antibodies elicited by these vaccines bound to Aβ monomers, oligomers, fibrils, and even APP [14, 15], also leading to cerebral edema and microvascular hemorrhage in the brains of patients with AD, and they did not show remarkably therapeutic effects in the clinical trials [16–18]. Passive immunotherapy using antibodies against Aβ monomers, such as bapineuzumab [19] and solanezumab [20], was also unsuccessful in AD clinical trials. However, aducanumab, an antibody recently developed by Biogen (Cambridge, MA, USA), selectively targeted aggregated Aβ, reduced Aβ levels in brains, and inhibited the clinical decline of recognition in patients with prodromal or mild AD in a phase I clinical trial. Aducanumab entered phase III clinical trials directly without a phase II clinical study [4]. Another phase III clinical study demonstrated that intravenous immunoglobulin (IVIG) exhibited beneficial effects on the subgroup of moderate and apolipoprotein E ε4 allele carrier patients with AD [21]. The antibodies against Aβ oligomers in IVIG were considered to contribute to these beneficial effects on AD treatment [22]. Consistently, our Aβ oligomer-specific antibodies (AβO) purified from IVIG (IVIG-AβO) attenuated the cognitive deficits and Aβ pathologies in APPswe/PS1dE9-transgenic mice [23]. These studies suggest that antibodies targeting Aβ oligomers may exert more efficient therapeutic effects on AD treatment. To generate a vaccine that induces antibodies to specifically neutralize Aβ oligomers, we first obtained Aβ_42_ oligomeric mimotopes by panning the phage-displayed random peptide libraries using IVIG-AβO as the target protein, then we expressed these mimotopes on EBY100 *Saccharomyces cerevisiae* to develop a novel Aβ oligomer-specific vaccine*.*

## Methods

### Materials

The Ph.D.™-12 Phage Display Peptide Library was obtained from New England Biolabs (Ipswich, MA, USA). Aβ_42_ was purchased from the American Peptide Company (Sunnyvale, CA, USA). IVIG-AβO were purified from IVIG by Aβ_42_ oligomer affinity chromatography at our laboratory [23]. EBY100 *S. cerevisiae* was a generous gift from Dr. Xiang-mei Liu, Shandong University, Jinan, China. Both Aβ_40_ and Aβ_42_ kits for Aβ measurement were purchased from Immuno-Biological Laboratories Co., Ltd. (Gunma, Japan). The following antibodies were used: 4G8 (monoclonal raised against Aβ_17–24_; Signet Laboratories/Covance Research Products, Denver, PA, USA), anti-ionized calcium-binding adaptor molecule-1 (anti-Iba-1) polyclonal antibody (GeneTex, Irvine, CA, USA), 9E10 (anti-c-Myc antibody; Santa Cruz Biotechnology, Dallas, TX, USA), anti-glial fibrillary acidic protein (anti-GFAP) monoclonal antibody (Cell Signaling Technology, Danvers, MA, USA), and anti-synaptophysin antibody (Abcam, Cambridge, UK). HRP-conjugated goat antimouse immunoglobulin G (IgG) antibody and HRP-conjugated goat antirabbit IgG antibody were obtained from Beijing Zhongshan Golden Bridge Biotechnology Co., Ltd. (Beijing, China). IL-4 and interferon (IFN)-γ enzyme-linked immunospot (ELISPOT) assay kits were purchased from Shenzhen DAKEWEI Co. Ltd. (Shenzhen, China). The iron stain kit we used was purchased from Sigma-Aldrich (St. Louis, MO, USA). The enhanced chemiluminescence (ECL) kit we used was purchased from Thermo Fisher Scientific (Waltham, MA, USA).

### Preparation of Aβ monomers, oligomers, and fibrils

Aβ_42_ (American Peptide Company) was dissolved in 100% hexafluoroisopropanol (HFIP) to a concentration of 1 mg/ml, sonicated in a water bath for 5 minutes, aliquoted into microcentrifuge tubes, vacuum-dried, and stored at −20 °C. Immediately prior to use, the HFIP-treated Aβ_42_ was dissolved in dimethyl sulfoxide (DMSO) to 2 mg/ml and diluted to 80 μM in 20 mM PBS buffer (monomer solution), pH 7.4, and then incubated at 37 °C. The states of incubated Aβ were checked using transmission electron microscopy and thioflavin T dye at different time points, and Aβ after 2 h and 24 h incubation was used as an oligomer and a fibril, respectively.

### Screening and identification of oligomeric mimotopes

The oligomeric mimotope peptides were obtained by screening peptide libraries through phage display as previously described [24]. The Ph.D.™-12 Phage Display Peptide Library was applied, and the oligomer-specific antibody IVIG-AβO was used as the target protein. After 4 rounds of selection, 44 positive clones were picked up and sequenced, and their sequences were analyzed by using the BLAST program for a homology search. Eight candidates were selected.

### Vaccine preparation

To increase the immunogenicity of the mimotope peptides, a DNA fragment encoding the mimotope peptides was inserted into a modified vector of pCTCON2 and transfected into EBY100 (*S. cerevisiae*) as previously described [25, 26]. The mimotope peptide was conjugated with a c-Myc tag in its C-terminus and linked to the C-terminus of the AGA2 protein with a linker. The displayed mimotope on the yeast cells was determined by flow cytometry and confocal microscope after probing with anti-c-Myc antibody and fluorescein isothiocyanate (FITC)-labeled secondary antibody. The EBY100 displaying Aβ_15_ was used as the positive control, and EBY100 yeast cells alone served as the negative control. All of the yeast cell-based vaccine was heat-inactivated (56 °C, 1 h) and stored at −80 °C until use.

### Mouse immunization

To screen the Aβ oligomer-specific vaccines, C57BL/6 J mice (female, 6 weeks of age, *n* = 3 for each group) were immunized with 6 × 10^7^ cells of yeast-based vaccines twice at biweekly intervals. To evaluate the therapeutic effects of the vaccine, APP/PS1 double-transgenic mice with a C57BL/6 J background were purchased from The Jackson Laboratory (Bar Harbor, ME, USA). The transgenic mice and their littermate wild-type (WT) control mice (males, 6 months of age) were maintained with access to food and water ad libitum in a colony room kept at 22 ± 2 °C and 50 ± 5% humidity under a 12-h/12-h light/dark cycle.

APP/PS1 mice (males, 6 months of age) were immunized with 6 × 10^7^ cells of EBY100 (*n* = 10), AOE1 (*n* = 10), and Aβ_15_ (*n* = 5) eight times at biweekly intervals. WT mice were immunized with PBS. Blood samples were taken at regular intervals, and sera were prepared and stored at −80 °C until further use. All experimental protocols were approved (reference number 15-LRT1) by the Tsinghua University Animal Care and Use Committee. Experiments were performed according to the U.S. Animal Welfare Act and the Public Health Service Policy on Humane Care and Use of Laboratory Animals of the National Institutes of Health.

### Antibody titer determination

An indirect enzyme-linked immunosorbent assay (ELISA) was used to measure the titers of vaccine-induced antibodies in sera against Aβ_42_ oligomers and mimotope peptides. Ninety-six-well ELISA plates were coated with Aβ_42_ oligomers (500 ng/well) or mimotope peptides (1 μg/well) overnight at 4 °C. After the plates were blocked with 3% (wt/vol) bovine serum albumin in PBS for 2 h at 37 °C, twofold serial dilutions of sera were added in triplicates and incubated for 2 h at 37 °C. The bound antibodies were detected by adding HRP-conjugated antimouse antibody and 3,3′,5,5′-tetramethylbenzidine substrate. The enzyme reaction was stopped by adding 2 M H_2_SO_4_, and the optical density (OD) at 450 nm was recorded by using a SpectraMax M5 microplate reader (Molecular Devices, Sunnyvale, CA, USA).

The levels of different IgG subclasses were also assayed by indirect ELISA. After incubation of sera (dilution to 1:100), HRP-conjugated antimouse IgG1, IgG2a, IgG2b, and IgG3 antibodies (Santa Cruz Biotechnology) were added to the plates in a 1:5000 dilution and incubated at 37 °C for 1 h. Then the OD values were detected as described above.

### Behavioral tests

Novel object recognition (NOR) and a Y-maze were applied to detect cognitive function in immunized mice as previously described [27]. The NOR test is based on the spontaneous tendency of mice to exhibit more interactions with a novel rather than a familiar object. In the habituation phase, each mouse was allowed to freely explore the open-field area (a white box 40 cm wide × 40 cm deep × 40 cm high) in the absence of objects. During the familiarization period, each mouse was placed in the box, which contained two identical objects, for 5 minutes. Recognition memory was tested after 24 h by exposing the mice to one familiar and one novel object. The time spent exploring and sniffing each object was recorded. The discrimination index was determined by performing the following calculation: (Time_novel_ − Time_old_)/(Time_novel_ + Time_old_).

After the NOR tests, the spatial recognition memory of the mice was tested by using the Y-maze test. Y-mazes were made of gray wood, covered with black paper, and consisted of three arms with an angle of 120 degrees between each arm. Each arm was 8 cm × 30 cm × 15 cm (width × length × height). The three identical arms were randomly designated as the start arm, in which the mouse started to explore (always open); the novel arm, which was blocked during the first trial but open during the second trial; and the other arm (always open). The Y-maze test consisted of two trials separated by an intertrial interval (ITI) to assess spatial recognition memory. The first trial (training) had a 10-minute duration and allowed the mouse to explore only two arms (start arm and other arm) of the maze, with the third arm (novel arm) being blocked. After a 1-h ITI, all three arms were accessible for the mice for the second trial. The mice were placed back in the maze in the starting arm, with free access to all three arms for 5 minutes. By using a ceiling-mounted charge-coupled device camera, all trials were recorded on a videocassette recorder, and the number of entries and time spent in each arm in the video recordings were analyzed.

### Dot blot analysis

Dot blot analysis was used to test the binding of vaccine-induced antibodies to various Aβ_42_ aggregates. Briefly, samples of Aβ_42_ monomers, oligomers, and fibrils were applied to the membranes and blocked at room temperature (RT) for 1 h with 5% nonfat milk. The membranes were then incubated at RT for 1 h with mouse sera diluted in PBS with Tween-20 (PBST), washed thrice for 10 minutes each, and incubated with relevant secondary antibodies in PBST for 1 h. The blots were washed thrice and developed with an ECL kit (Pierce Biotechnology, Rockford, IL, USA).

### Western blot analysis

7PA2 cell (CHO cells stably transfected with a complementary DNA-encoding APP751 that contains the Val717Phe familial AD mutation) lysates were used to determine the binding capacity of vaccine-induced antibodies to various Aβ aggregates. The cell extracts mixed with Aβ monomers were boiled together with 5× loading buffer in the presence of dithiothreitol (DTT) for 10 minutes and electrophoresed on a 4–12% SDS-PAGE gel, and then the gel was transferred onto a nitrocellulose (NC) membrane. The membrane was probed with 4G8 and vaccine-induced antibodies, followed by HRP-conjugated antimouse IgG, and then developed with an ECL kit.

To determine the change of Aβ oligomers in the AD mouse brains after vaccination, brain extracts were boiled together with 5× loading buffer in the presence of DTT for 10 minutes and loaded onto a 4–12% SDS-PAGE gel. The separated proteins were transferred onto NC membranes, which were then washed and incubated with 4G8 overnight at 4 °C. The membranes were washed again and incubated with HRP-conjugated secondary antibody for 1 h at RT. The blots were developed with an ECL kit according to the manufacturer’s instructions. The intensity of protein bands was quantified using ImageJ software (National Institutes of Health, Bethesda, MD, USA).

### Cerebral homogenate collection

After the behavioral tests, the mice were immediately intraperitoneally anesthetized with avertin (300 mg/kg), perfused with ice-cold PBS containing heparin (10 U/ml), and then killed. The brain was rapidly removed and divided. One hemisphere was rapidly dissected and homogenized. The brain tissues were Dounce-homogenized in radioimmunoprecipitation assay (RIPA) buffer containing a protease inhibitor mixture, which consisted of 50 mM Tris (pH 7.4), 150 mM NaCl, 1% Triton X-100, 1% sodium deoxycholate, and 0.1% SDS. The tissues were then centrifuged at 14,000 × *g* for 30 minutes at 4 °C, and the supernatant (RIPA-soluble fraction) containing soluble Aβ was collected. The pellets were resuspended in guanidine buffer (5.0 M guanidine-HCl/50 mM Tris-HCl, pH 8.0) and centrifuged at 14,000 × *g* for 1 h at 4 °C to obtain supernatants containing insoluble Aβ (guanidine-soluble Aβ).

### Measurement of Aβ_40/42_

To determine the levels of Aβ in the brain, RIPA-soluble and RIPA-insoluble (guanidine-soluble) Aβ fractions of mice were quantified by ELISA using Aβ_40_ and Aβ_42_ immunoassay kits according to the manufacturer’s instructions. The levels of soluble and insoluble Aβ were standardized to the brain tissue weight and expressed in micrograms of Aβ per gram of brain tissue.

### Immunohistochemistry

Immunohistochemical staining was performed as previously described [28]. Briefly, 20-μm-thick sections at intervals of 100 μm were obtained using a freezing microtome (Leica Microsystems, Wetzlar, Germany) and mounted on poly-l-lysine-coated slides. The sections were washed in PBS and then treated briefly with 80% (vol/vol) methanol containing 0.3% H_2_O_2_ to prevent endogenous peroxidation. The sections were then blocked with 10% normal goat serum to prevent nonspecific protein binding. Subsequently, the sections were incubated with the primary antibodies 6E10 (1:100), GFAP (1:100), Iba-1 (1:100), and synaptophysin (1:100) for 1 h at RT, followed by incubation with an HRP-labeled or fluorescence-labeled secondary antibody at RT for 1 h. The targets were visualized with 3,3′-diaminobenzidine substrate and counterstained with hematoxylin. Images were collected using an Olympus BX60 microscope (Olympus Optical Co. Ltd., Tokyo, Japan) by using × 4 and × 10 lens objectives. IpWin5 analytical software was used to quantify the glial cells.

### Analysis of microhemorrhage

To assess the number of microhemorrhages, sections were stained using the iron stain kit (Sigma-Aldrich) according to the manufacturer’s protocol. Three slides per animal were analyzed. Prussian blue-positive spots in subregions of the cortex as well as in the hippocampus were manually counted.

### ELISPOT assay

One week after the behavioral tests, three mice of each group were killed, and their splenocytes were isolated and analyzed for the presence of target-specific T cells by ELISPOT analysis according to the manufacturer’s protocol. Full-length Aβ_1–42_ (10 mg/ml), L2 peptides (10 mg/ml), EBY100 (10^5^ cells/ml), negative control DMSO and PBS, and positive control phorbol 12-myristate 13-acetate (PMA) and ionomycin were used for splenocyte restimulation. The levels of interleukin 4 (IL-4) or IFN-γ were measured to evaluate T-cell stimulation.

### Statistical analysis

Data were obtained from at least three independent experiments for each condition and are expressed as the mean ± SEM. Statistical significance was analyzed using Student’s *t* test.

## Results

### Selection and expression of Aβ oligomer-specific mimotopes

**Fig. 1:**
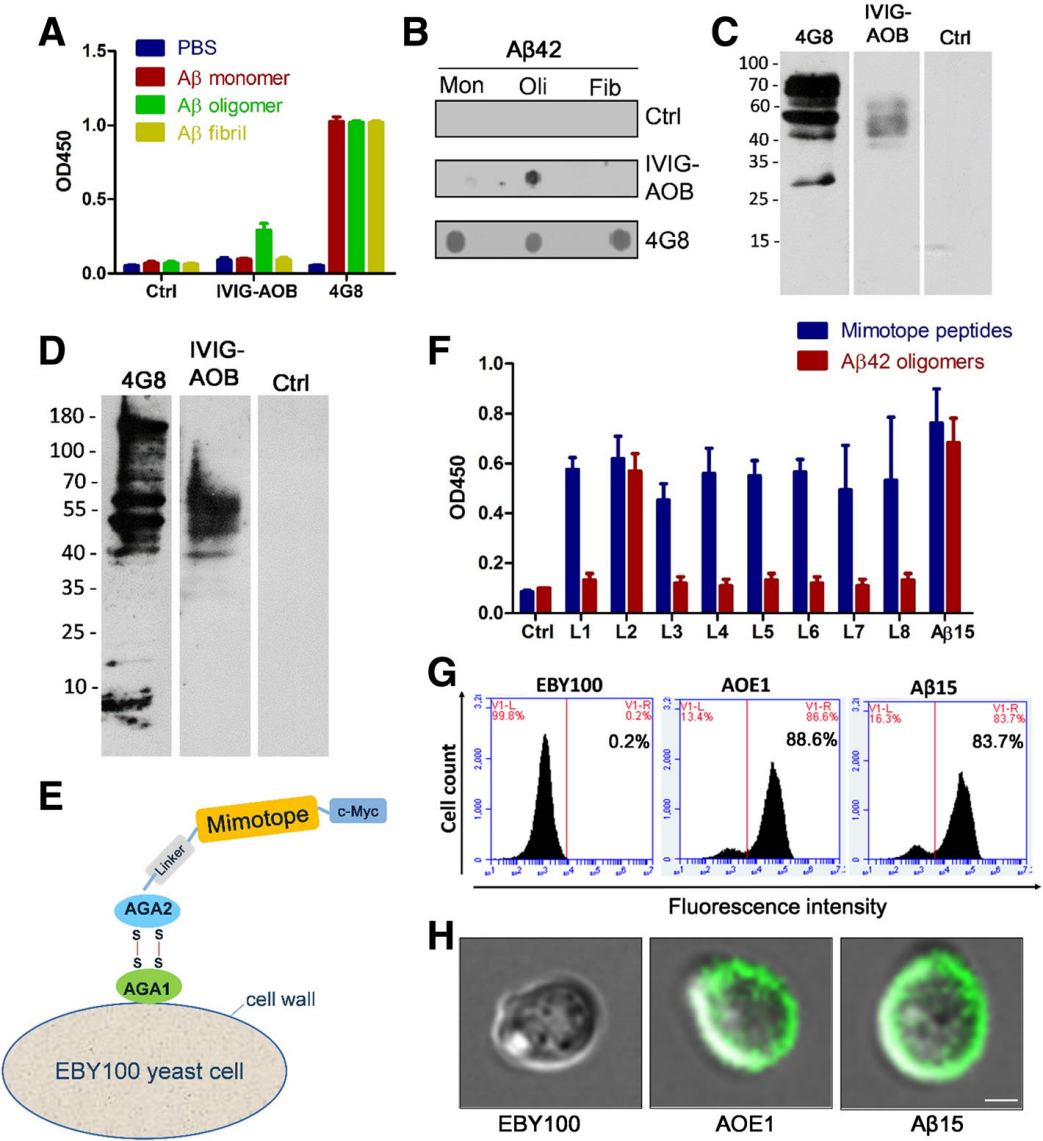
Selection and expression of β-amyloid peptide (Aβ) oligomer-specific mimotopes. **a–d** Aβ oligomer-specific antibodies purified from intravenous immunoglobulin G (IVIG-AβO) specifically recognized Aβ oligomers. The binding of IVIG-AβO to different Aβ forms was detected by enzyme-linked immunosorbent assay (ELISA) (**a**) and dot blot analysis (**b**), respectively. The brain homogenate of APP/PS1 mice (**c**) and 7PA2 cell lysates mixed with Aβ monomer (**d**) were separated on SDS-PAGE gels, and then Western blot analysis was performed with IVIG-AβO as primary antibodies. An irrelevant human monoclonal antibody and 4G8 were used as negative and positive controls, respectively. **e** Structural diagram of yeast-based vaccine. The mimotopes with c-Myc were linked with the C-terminal of AGA2 protein and displayed on the surface by interaction with AGA1. **f** C57BL/6 mice (female, 6 weeks of age, *n* = 3 for each group) were subcutaneously injected twice at a 2-week interval with eight candidate yeast-based vaccines in the absence of adjuvant. The antibody titers against mimotope peptides and Aβ_42_ oligomers in the mice immunized with different vaccines were detected by ELISA. One vaccine, EBY100-L2, was termed *AOE1*. **g** The AOE1 and Aβ_1–15_ were expressed and displayed on the surface the EBY100 yeast cells. The cells were probed with anti-Myc antibody and fluorescein isothiocyanate-labeled secondary antibody and then analyzed by flow cytometry. EBY100 yeast cells were used as a negative control. **h** Expression of AOE1 and Aβ_1–15_ was determined by confocal microscopy. The obvious fluorescent signals were detected on the surface of EBY100 yeast cells expressing L2 and Aβ_1–15_. Bar indicates 1 μm. *OD450* Optical density at 450 nm

To construct a vaccine specifically against Aβ oligomers, we first isolated Aβ oligomeric mimotopes by screening the Ph.D.™-12 Phage Display Peptide Library using IVIG-AβO. The binding characteristics of the purified IVIG-AβO were detected by ELISA, dot blot analysis, and Western blot analysis. The results indicated that IVIG-AβO specifically recognized Aβ oligomers rather than Aβ monomers of fibrils (Fig. 1a–d). After four rounds of selection, positive clones were picked up and sequenced, and their sequences were analyzed by using the BLAST program for a homology search. Eight candidate epitope peptides (Table 1) were expressed and displayed on EBY100 *S. cerevisiae* (Fig. 1e) to generate whole-cell-based vaccines. EBY100 displaying the Aβ_1–15_ fragment was used as a vaccine control. To test the ability to elicit antibodies against Aβ_42_ oligomers, nine yeast-based vaccines were subcutaneously injected into C57BL/6 mice two times. One vaccine, EBY100-L2 (termed *AOE1*), could elicit anti-Aβ_42_ oligomeric antibodies, whereas other vaccines induced antibodies only against mimotope peptides rather than Aβ_42_ oligomers (Fig. 1f).

**Table 1:** Sequences of eight candidate mimotopes

Mimotope	Sequence
L1	RTTELEPYENEQ
L2	RPDQVMWDSKRP
L3	FLRMDHSALGSV
L4	TRSWLRTQWWIR
L5	TPSIGPNSTWLD
L6	SVWSYWHSSHRH
L7	HFYQAQKTTWAS
L8	NFSGEHSSRNRP

To further validate the exhibition of mimotope peptide L2 on the EBY100 surface, the yeast cells were incubated with anti-Myc antibody and then reacted with secondary antibody labeled with FITC for detection by flow cytometry and confocal microscopy. The yeasts expressing L2 and Aβ_1–15_ showed obvious signals in comparison to EBY100 yeast cell controls (Fig. 1g). More than 80% of the yeast cells were signal-positive in both groups expressing L2 or Aβ_1–15_. Moreover, the confocal microscopy results also indicated that L2 and Aβ_1–15_ peptides were displayed on the surface of the yeast (Fig. 1h), whereas no green fluorescence was detected on the EBY100 yeast cell controls.

### Immunogenicity of AOE1

**Fig. 2:**
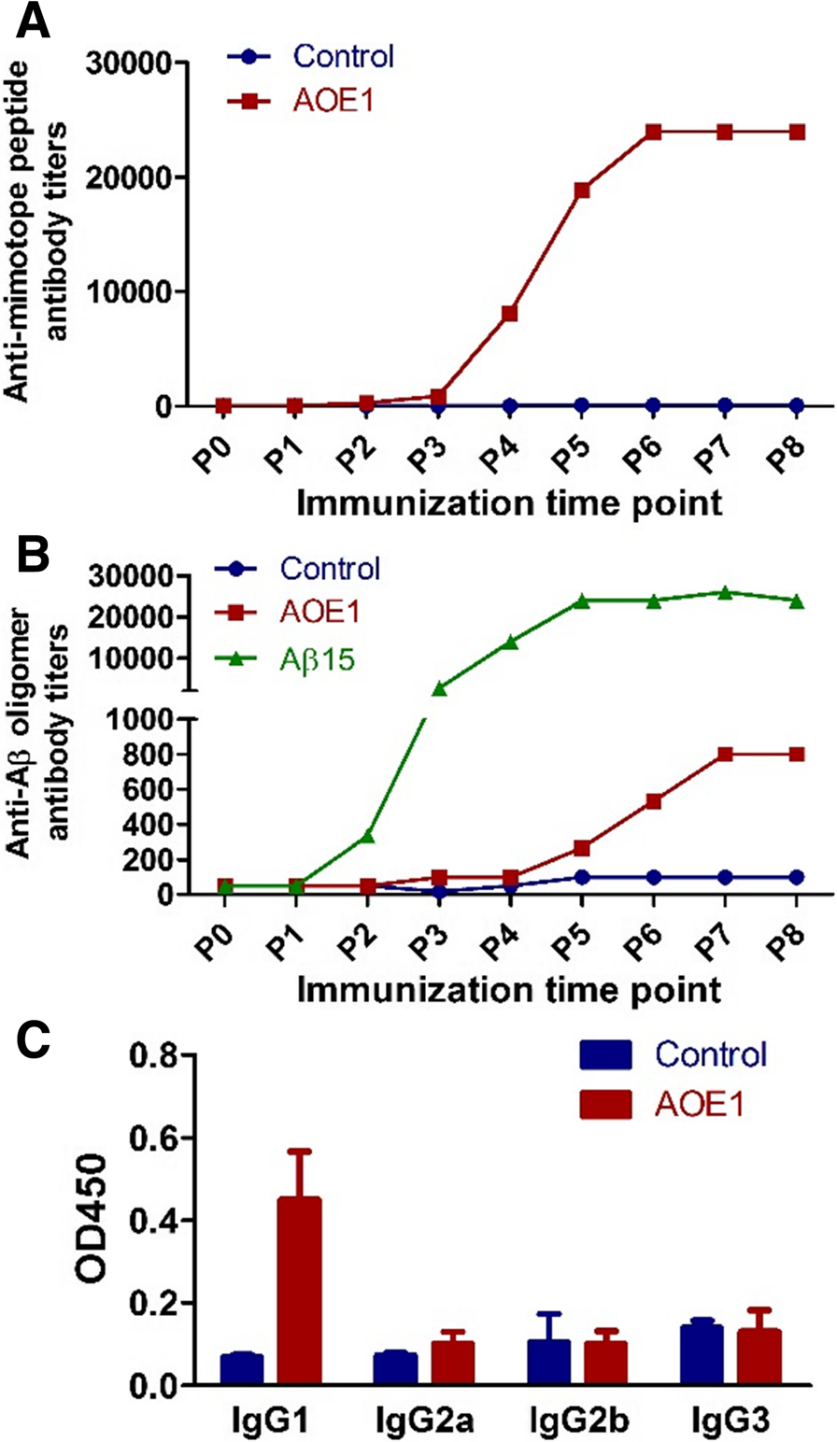
The titer and subtype of antibody in Alzheimer’s disease (AD) transgenic mice immunized with EBY100-L2 vaccine (AOE1). APP/PS1 mice (male, 6 months of age) were subcutaneously injected eight times at 2-week intervals with AOE1, Aβ_15_, and EBY100 control cells in the absence of adjuvant. The titers and subtypes of antibody in the mouse sera were detected by enzyme-linked immunosorbent assay. **a** The titers of antibody against mimotope peptide L2 in the mice immunized with AOE1 vaccine. **b** The titers of antibody against Aβ_42_ oligomers in the mice immunized with AOE1 vaccine. **c** Subtype of immunoglobulin G against Aβ_42_ oligomers. *OD450* Optical density at 450 nm

To test the immunogenicity of AOE1, APP/PS1 mice were injected subcutaneously eight times at 2-week intervals with a dose of 6 × 10^7^ cells in the absence of adjuvant. EBY100 yeast cells were used as negative controls. The antibody titers were measured with synthetic mimotope peptides and Aβ oligomers as the antigens, respectively. After the sixth immunization, AOE1 induced a high antibody titer against mimotope peptide to 1:24,000, whereas no antibody was detected in the control group (Fig. 2a). Moreover, the antibody induced by AOE1 recognized Aβ oligomers, and its titer reached 1:800 (Fig. 2b). To determine the isotypes of the anti-Aβ oligomer IgG induced by AOE1, sera from the mice were diluted (1:100) and tested by indirect ELISA. The results indicated that IgG1 was the predominant form of the antibody, whereas IgG2a, IgG2b, and IgG3 were detected at low levels, suggesting that AOE1 induced a noninflammatory type 2 helper T-cell immune response (Fig. 2c).

### Specificity of the antibody induced by AOE1

**Fig. 3:**
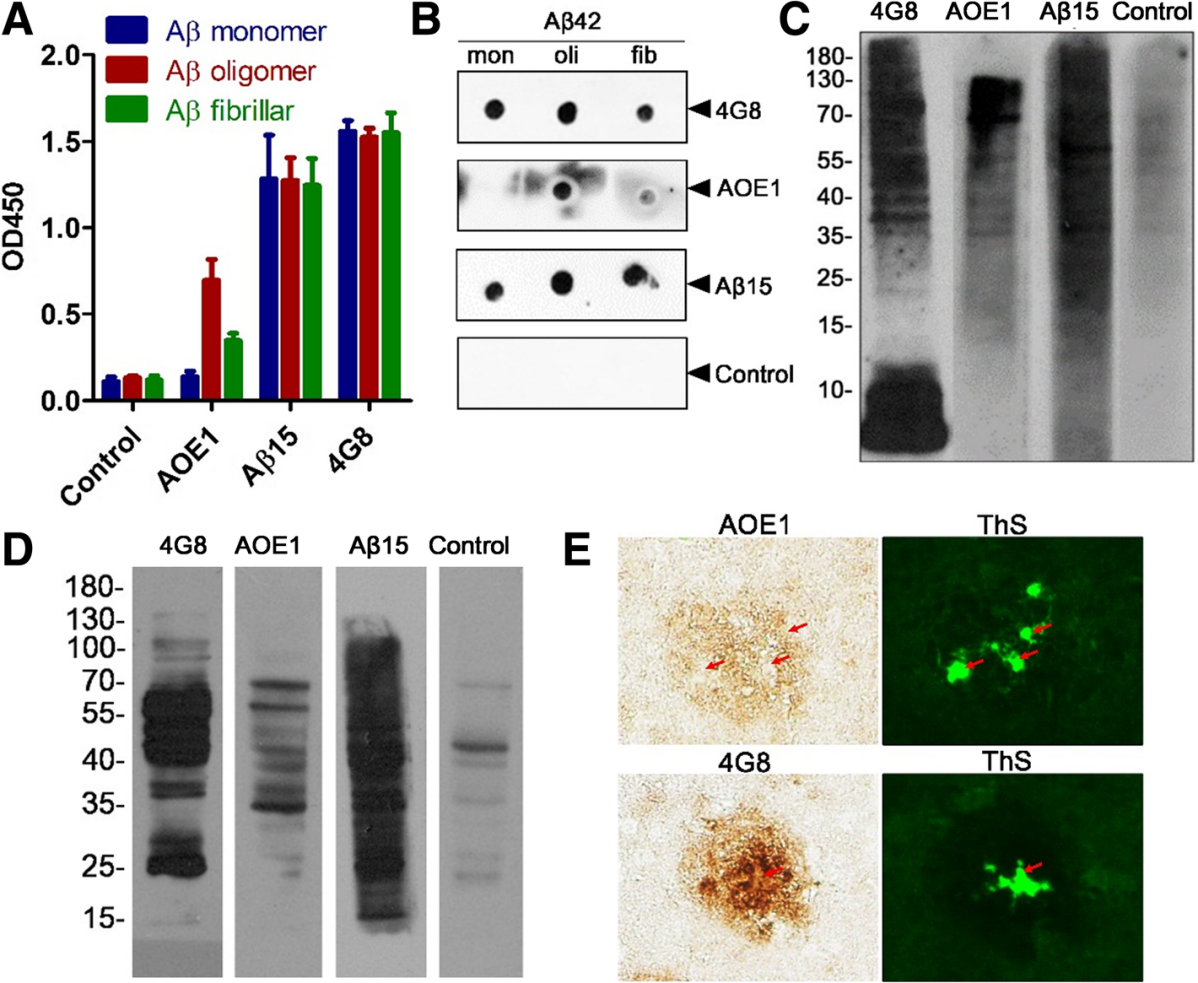
The antibody induced by EBY100-L2 vaccine (AOE1) specifically recognized β-amyloid peptide (Aβ) oligomers. After the last immunization, the sera from the mice immunized with AOE1 vaccine or Aβ_15_ were diluted to 1:100 or 1:1000, respectively. The binding of antibodies induced by AOE1 vaccine or Aβ_15_ to different Aβ isoforms was detected by enzyme-linked immunosorbent assay (**a**) or dot blot analysis (**b**), respectively. 7PA2 cell lysates mixed with Aβ monomer (**c**) and brain homogenate of APP/PS1 mice (**d**) were separated on SDS-PAGE gels, and then Western blot analysis was performed with sera from vaccine-immunized mice as primary antibodies. **e** Brain sections from APP/PS1 mice were incubated with sera of AOE1-treated mice and 4G8 and then stained with thioflavin S (ThS). *Red arrows* indicate the ThS-positive compact plaques. *OD450* Optical density at 450 nm

To further determine the specificity of the antibody induced by AOE1, Aβ monomers, oligomers, and fibrils were coated onto ELISA plates and incubated with the sera of mice vaccinated with AOE1. The results indicated that the antibody induced by Aβ_1–15_ vaccine, just like the anti-Aβ monomer antibody 4G8, bound to all forms of Aβ, whereas the AOE1-elicited antibodies specifically bound to Aβ oligomers rather than to Aβ monomers, and also bound Aβ fibrils with low affinity (Fig. 3a). Consistent with our ELISA results, our dot blot analysis results also showed that AOE1-induced sera strongly bound to Aβ oligomers but not to monomers, although Aβ_1–15_-induced sera and 4G8 recognized all forms of Aβ (Fig. 3b). These results indicated that AOE1 vaccine stimulated mainly the production of oligomer-specific antibodies.

To confirm the size of Aβ oligomers to which AOE1 induced sera bound, Western blot analysis was performed using 7PA2 cell lysates mixed with Aβ_42_ monomers or brain homogenates of APP/PS1 mice as antigens. Aβ_15_-stimulated sera and 4G8 antibody bound to all sizes of Aβ, whereas sera induced by AOE1 bound to Aβ oligomers from a range of 35–100 kDa (Fig. 3c, d), which was consistent with the binding of IVIG-AβO to Aβ.

To determine whether the AOE1-induced antibody bound to endogenous Aβ plaques, the brain sections of APP/PS1 mice were stained by performing immunohistochemistry (IHC) using AOE1-induced sera with 4G8 as a positive control. The results showed that 4G8 strongly bound to both the diffuse and compact regions of plaques, but AOE1-induced sera selectively bound to loose rather than compact regions, which were remarkably stained by thioflavin S (ThS) (Fig. 3e), consistent with previous reports that the dense cores of plaques are composed mainly of Aβ fibrils and few oligomers [29]. These results further confirmed that AOE1-induced sera recognized mainly Aβ oligomers.

### AOE1 vaccination attenuates cognitive impairment in APP/PS1 mice

**Fig. 4:**
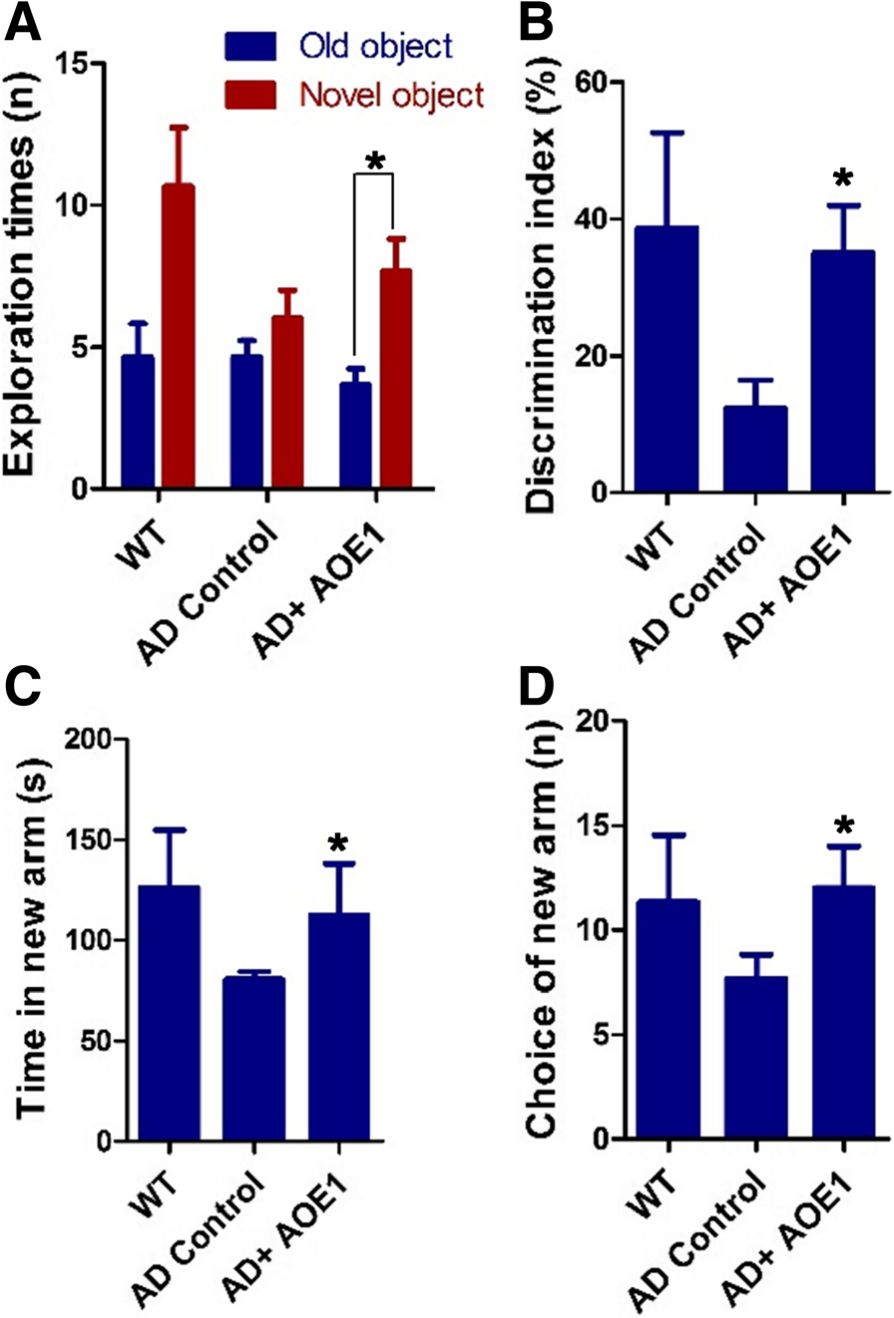
EBY100-L2 (AOE1) vaccination attenuated cognitive deficits in APP/PS1 transgenic mice. The cognitive function of Alzheimer’s disease (AD) mice was determined by novel object recognition (NOR) and Y-maze tests 2 weeks after the last vaccination. **a** Exploration times of mice for the old and new objects during NOR tests (compared with exploration times for old object, *p < 0.05). **b** Discrimination index of mice for the new object during the NOR test (compared with AD control, *p < 0.05). **c** and **d** Time spent in the new arm (**c**) and the entries into the new arm (**d**) of mice vaccinated with or without AOE1 vaccine (compared with AD control, *p < 0.05). *WT* Wild type

NOR and Y-maze tests were conducted to evaluate the effect of AOE1 vaccination on cognitive function in APP/PS1 transgenic mice. During the probe trial of the NOR test, the exploration times for the novel object among AOE1-treated mice were significantly increased compared with the old object, whereas the control-treated mice exhibited similar exploration times for recognizing both novel and old objects (*t* test, *p* = 0.005, *n* = 10) (Fig. 4a). The discrimination index of AOE1-treated mice showed a marked increase compared with that of the control-treated mice (*t* test, *p* = 0.008, *n* = 10) (Fig. 4b). In the Y-maze test, AOE1-treated mice spent more time in the new arm and entered into the new arm more times than the control-treated mice (*t* test; for time in new arm, *p* = 0.04, *n* = 10; for choice of new arm, *p* = 0.03, *n* = 10) (Fig. 4c, d). Taken together, these results indicated that AOE1 vaccine treatment attenuated cognitive impairment in AD transgenic mice.

### AOE1 vaccination lowers cerebral Aβ levels in AD transgenic mice

**Fig. 5:**
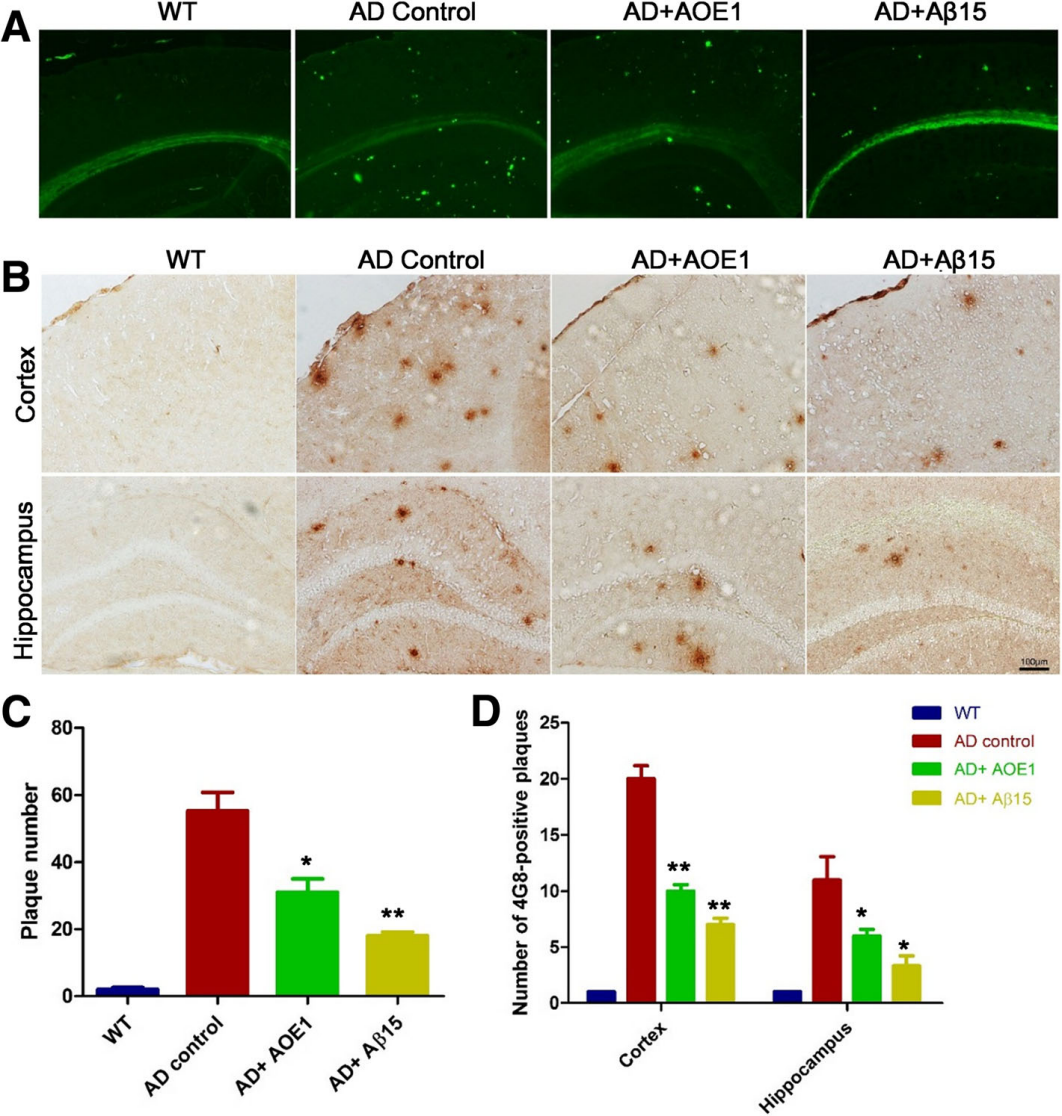
EBY100-L2 (AOE1) immunization decreased β-amyloid peptide (Aβ) deposits in the brains of Alzheimer’s disease (AD) mice. The plaques in the mouse brains were detected by thioflavin S (ThS) staining (**a**) and immunohistochemistry (IHC) using 4G8 (**b**), and the number of plaques in the brain sections stained by ThS staining (**c**) and IHC (**d**) was quantified using IpWin5 software (compared with AD control, *p < 0.05, **p < 0.01). *WT* Wild type

To investigate the effects of AOE1 vaccine on the Aβ burden in the brains of AD transgenic mice, we detected Aβ deposits by ThS staining and IHC using 4G8 antibody. No Aβ deposits were detected in the brain tissues of WT mice. The AD mouse controls formed robust senile plaques in the cortex and hippocampus, whereas the plaque numbers in brain sections of AOE1- or Aβ_15_-treated mice decreased (*t* test; ThS staining, *p* < 0.05 for AOE1, *p* < 0.01 for Aβ_15_, *n* = 7; 4G8 IHC, *p* < 0.01 for cortex and *p* < 0.05 for hippocampus of AOE1 and Aβ_15_, *n* = 7) (Fig. 5).

**Fig. 6:**
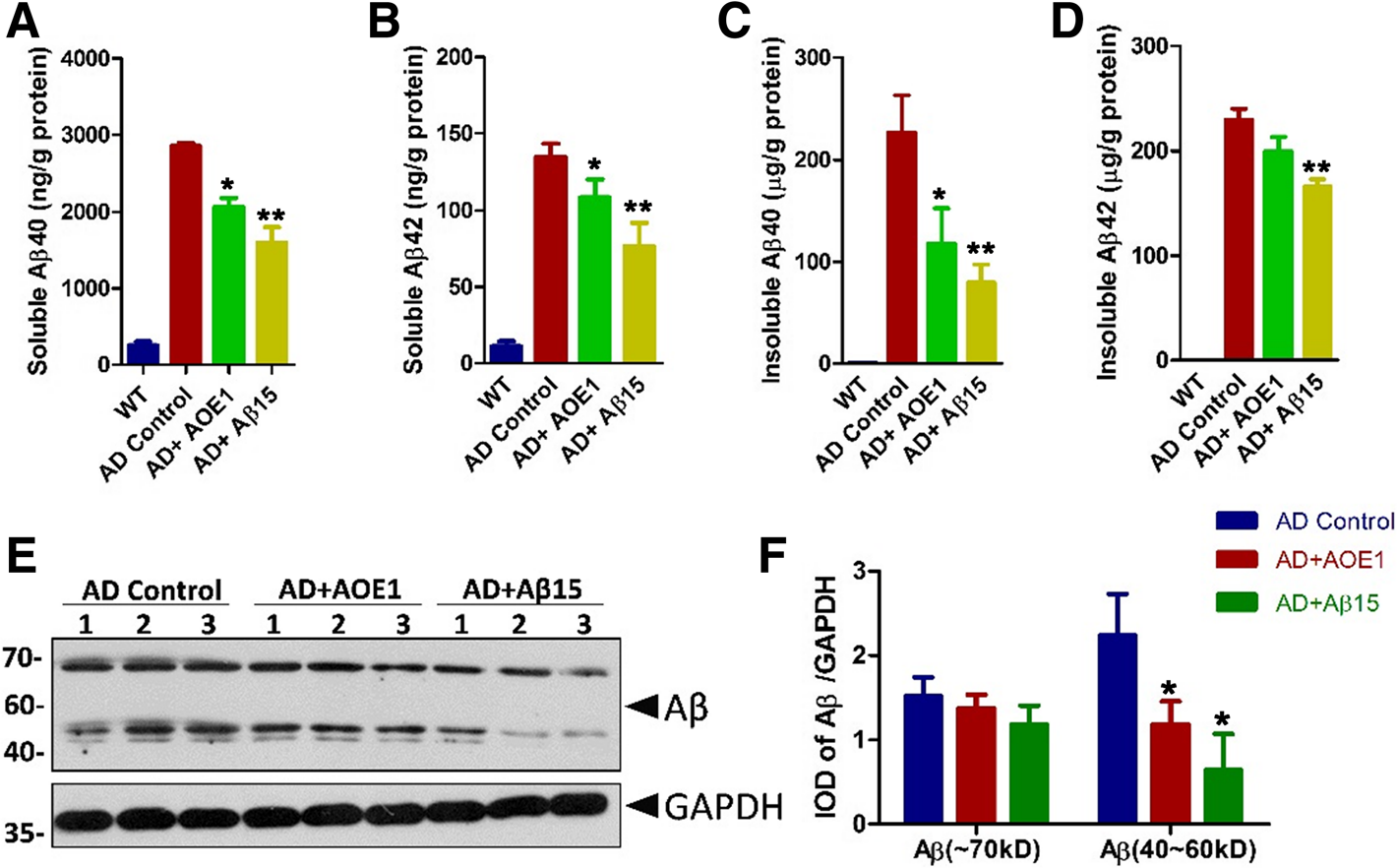
EBY100-L2 (AOE1) immunization reduced β-amyloid peptide (Aβ) levels in the brains of Alzheimer’s disease (AD) mice. After the behavior test, the mouse brain homogenate was prepared by using radioimmunoprecipitation assay buffer (soluble fraction) and guanidinium chloride buffer (insoluble fraction), respectively. Soluble (**a**, **b**) and insoluble (**c**, **d**) Aβ_40_ (**a**, **c**) and Aβ_42_ (**b**, **d**) were determined using an Aβ enzyme-linked immunosorbent assay kit (**a–d**) (compared with AD control, *p < 0.05, **p < 0.01). **e** The Aβ oligomer levels in the brain extracts of mice immunized with or without AOE1 were detected by Western blot analysis, and the band density was quantified using IpWin5 software (**f**) (compared with AD control, *p < 0.05). *GAPDH* Glyceraldehyde 3-phosphate dehydrogenase, *WT* Wild type, *IOD* Integrated optical density

Further, we detected the levels of soluble and insoluble Aβ in the mouse brains by ELISA. Compared with levels in AD control mice, the levels of soluble and insoluble Aβ_40_ and soluble Aβ_42_ in AOE1- or Aβ_15_-treated AD mice significantly decreased (*t* test; soluble Aβ_40_, *p* < 0.05 for AOE1, *p* < 0.01 for Aβ_15_, *n* = 7; insoluble Aβ_40_, *p* = 0.048 for AOE1, *p* = 0.003 for Aβ15, *n* = 7; soluble Aβ_42_, *p* = 0.03 for AOE1, *p* = 0.004 for Aβ15, *n* = 7) (Fig. 6a–c). AOE1 immunization also decreased the insoluble Aβ_42_ level, but without statistical significance (*t* test, *p* = 0.10, *n* = 7) (Fig. 6d), whereas Aβ_15_ significantly reduced the insoluble Aβ_42_ level (*t* test, *p* < 0.01, *n* = 7) (Fig. 6d).

Soluble Aβ oligomer levels are related to cognitive decline in patients with AD [30]. To test whether AOE1 vaccination reduced the levels of Aβ oligomers, the mouse brain extracts were explored by Western blot analysis. AOE1 or Aβ_15_ immunization significantly decreased the levels of Aβ oligomers with a molecular weight range from 40 to 60 kDa (*t* test, *p* = 0.046 for AOE1, *p* = 0.014 for Aβ15, *n* = 3) (Fig. 6e, f), whereas the levels of oligomers with a molecular weight around 70 kDa did not change (Fig. 6e, f).

### AOE1 vaccination suppresses activation of glia in brains of AD mice

**Fig. 7:**
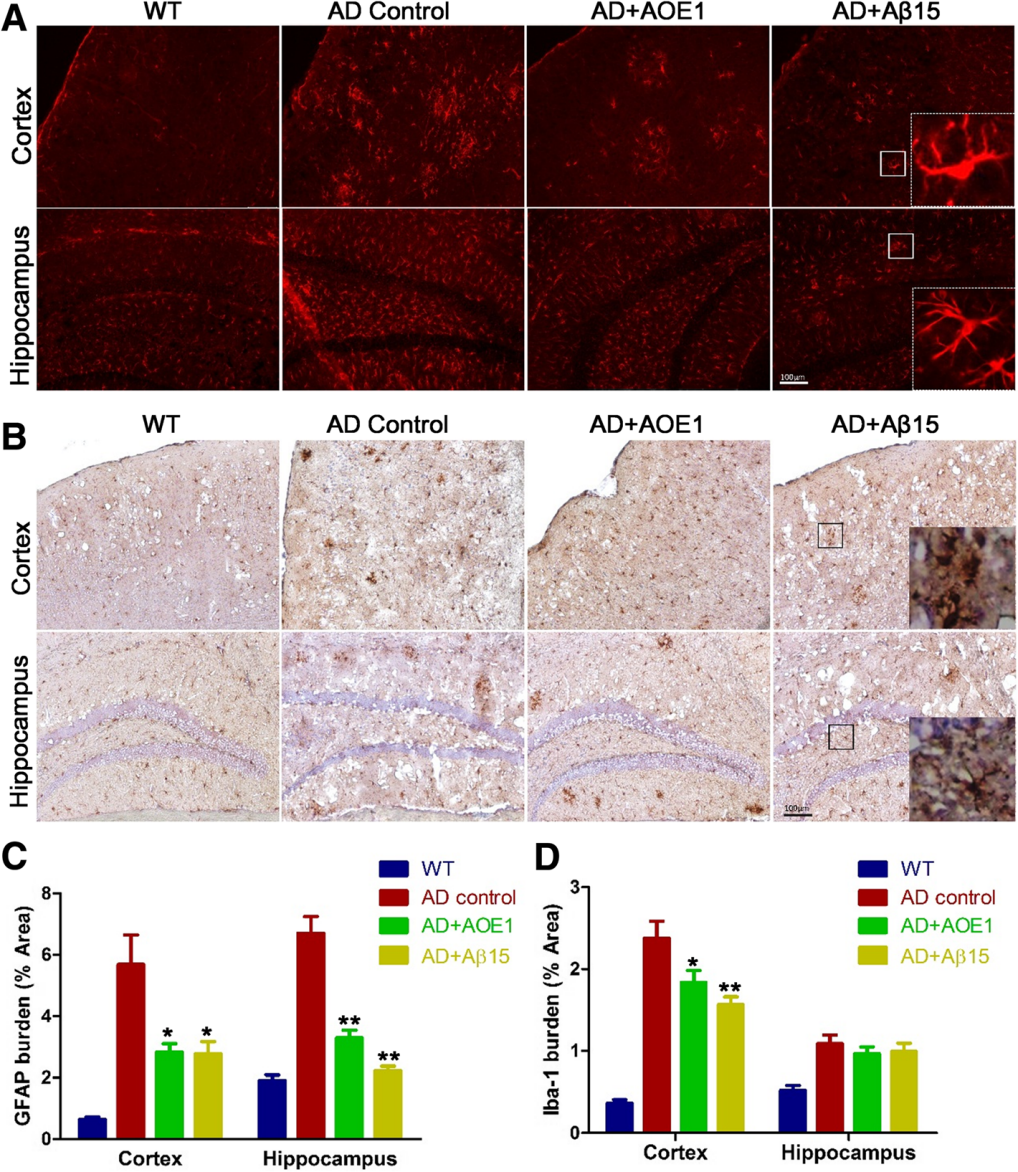
EBY100-L2 (AOE1) vaccination decreased activated astrocytes and microglia in the hippocampus and cortex of Alzheimer’s disease (AD) mice. **a** Astrocytes in the cortex and hippocampus of brain slices were determined by immunohistochemistry (IHC) using glial fibrillary acidic protein (GFAP) monoclonal antibody, and then the stained astrocytes were quantified using IpWin5 software (**c**) (compared with AD control, *p < 0.05, **p < 0.01). **b** Microglia in the cortex and hippocampus of brain slices were detected by IHC using ionized calcium-binding adaptor molecule-1 (Iba-1) polyclonal antibody, and then the stained microglia were quantified using IpWin5 software (**d**) (compared with AD control, *p < 0.05, **p < 0.01). *Aβ* β-Amyloid peptide, *WT* Wild type

Because neuroinflammation plays a major role in the pathogenesis of AD, we next examined the effect of AOE1 treatment on the activation of astrocytes and microglia in the brains of mice with AD. To detected astrocyte activation, the brain slices were incubated with GFAP antibody and then probed with fluorescence-labeled secondary antibodies. In comparison with WT mice, a higher number of GFAP-immunopositive astrocytes were observed in the hippocampus and cortex of mice with AD, but the number significantly decreased after AOE1 or Aβ_15_ treatment (*t* test; cortex, *p* = 0.02 for AOE1, *p* = 0.02 for Aβ_15_, *n* = 7; hippocampus, *p* < 0.001 for AOE1, *p* < 0.001 for Aβ15, *n* = 7) (Fig. 7a and c). Anti-Iba-1 antibody was used to determine the activation of microglia. The results showed that the number of activated microglia in the cortex of AD mice was sharply decreased after AOE1 vaccination (*t* test; cortex, *p* < 0.01 for AOE1, *p* < 0.01 for Aβ_15_, *n* = 7; hippocampus, *p* = 0.16 for AOE1, *p* = 0.53 for Aβ_15_, *n* = 7) (Fig. 7b and d). These results indicated that AOE1 treatment suppressed the activation of astrocytes and microglia in vivo.

### AOE1 vaccination does not activate Aβ-specific T cells and trigger microhemorrhages

**Fig. 8:**
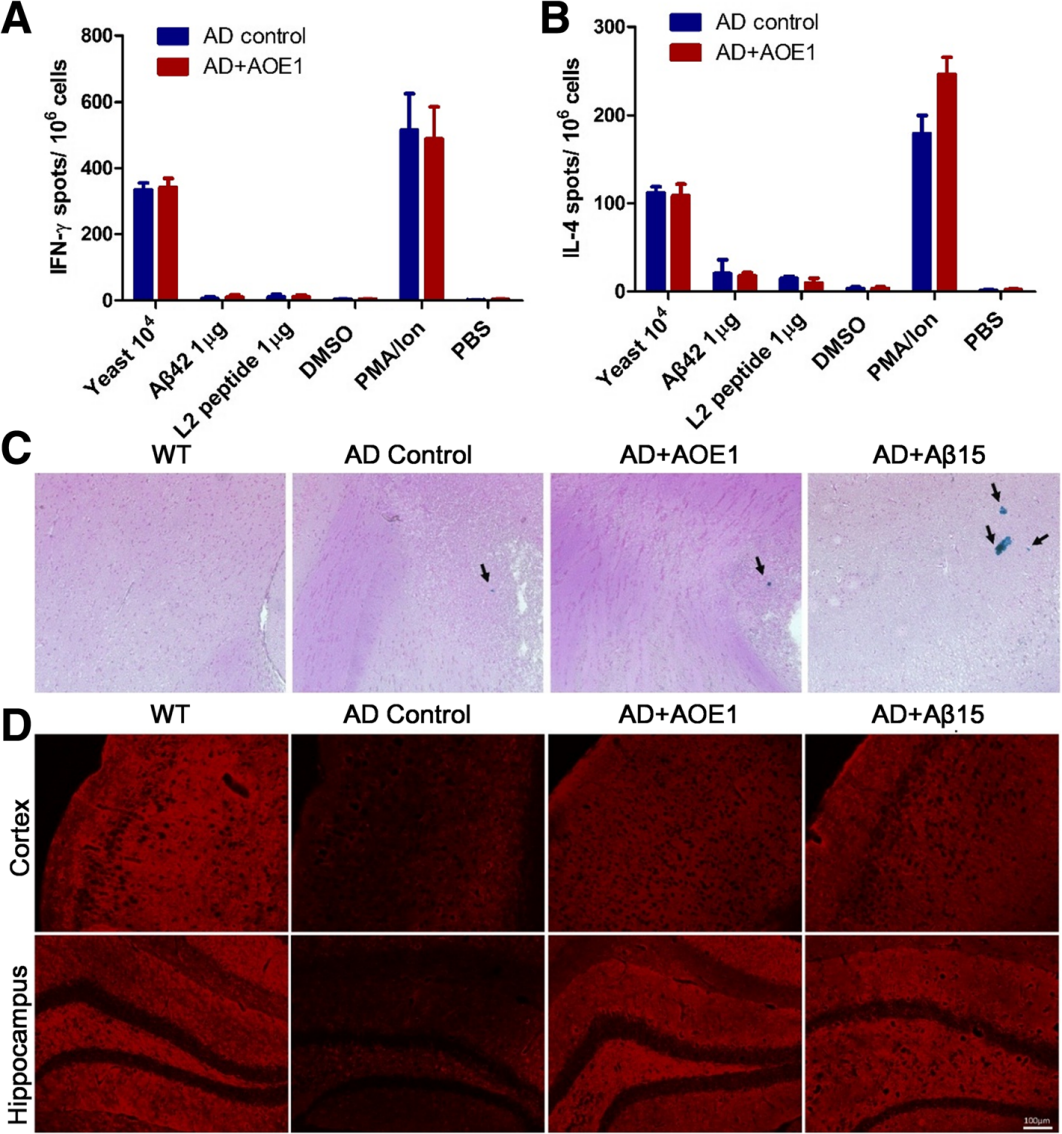
EBY100-L2 (AOE1) immunization does not activate β-amyloid peptide (Aβ)-reactive T cells and rarely induces microhemorrhages. T-cell activation in Alzheimer’s disease (AD) mice vaccinated with AOE1 was detected by enzyme-linked immunospot (ELISPOT) assay. The spleen cells were obtained from the AD mice vaccinated with AOE1 and restimulated with yeast carrier, Aβ_42_, AOE1 epitope peptides, and phorbol 12-myristate 13-acetate (PMA)/ionomycin (positive stimulator) as well as with dimethyl sulfoxide (DMSO) and PBS (negative control). The secretion of interferon (IFN)-γ (**a**) and interleukin (IL)-4 (**b**) were determined by ELISPOT assay. **c**, Microhemorrhages in the brain slices were determined through Prussian blue staining. Rare microhemorrhages were observed in the brains of mice vaccinated with AOE1. *Arrows* indicate microhemorrhage spots. **d** The synaptic pathology in the cortex and hippocampus of brain slices was determined through immunohistochemistry using antisynaptophysin antibody. *WT* Wild type

Previous reports demonstrated that Aβ vaccination might induce a T-cell-dependent immune response, resulting in meningoencephalitis and cephaledema. To explore the effect of AOE1 vaccination on the activation of Aβ-specific T cells, we measured the levels of IFN-γ and IL-4 by ELISPOT assay using the splenocytes of AD mice immunized with or without AOE1. The cultured splenocytes were stimulated with EBY100 yeast cells, Aβ_42_ peptide, and L2 peptide, respectively, with DMSO and PBS used as the negative controls and PMA/ionomycin [31] used as the positive control. PMA/ionomycin increased the production of IFN-γ and IL-4 by the splenocytes of AD mice immunized with both AOE1 vaccine and EBY100 yeast cells (Fig. 8a and b). However, restimulation by Aβ_42_ or L2 did not induce the increased secretion of IFN-γ and IL-4 in the splenocytes. These results indicated that AOE1 vaccination did not induce an Aβ-specific T-cell response.

Another side effect induced by previous Aβ vaccine is microhemorrhages [32]. To assess the microhemorrhages induced by AOE1 and Aβ_15_ vaccination, we stained brain sections with Prussian blue stain. Like AD control and WT mice, AOE1-immunized AD mice displayed rare microhemorrhages in the brain slides, whereas more microhemorrhages were observed in Aβ_15_-immunized AD mice. These results suggested that AOE1 might be a safer vaccine than Aβ_15_.

The effects of AOE1 or Aβ_15_ immunization on synaptic pathology in AD mice were assessed by immunostaining with antisynaptophysin antibodies (Fig. 8d). APP/PS1 control mice showed a significant decrease in synaptophysin fluorescence in the cortex and hippocampus, whereas immunization with AOE1 or Aβ_15_ significantly increased synaptophysin levels, indicating that AOE1-mediated Aβ oligomer clearance reduced synaptic pathology.

## Discussion

Aβ immunotherapy including active immunization with Aβ and passive immunization with anti-Aβ antibodies for AD treatment has been investigated for almost 2 decades [10]. However, none of the Aβ vaccines or antibodies were successfully developed to rescue the memory deficits of patients with AD in phase III clinical trials [32, 33]. The first-generation Aβ vaccine AN1792 was terminated in phase II clinical trials because of serious Aβ-specific T-cell reactions. To overcome this side effect, the second-generation Aβ vaccines, such as CAD-106, ACC-001, ACI-24, and MER5101, applied the N-terminal fragments of Aβ_42_ as immunogens [13, 34–36]. These vaccines elicited antibodies against Aβ monomers, oligomers, fibrils, and APP, but they still induced side effects such as cephaledema, autoimmune reactions and inhibition of the physiological functions of Aβ monomer and APP. Moreover, the therapeutic effects of the antibodies induced by the vaccines could be reduced by APP or Aβ monomer-induced neutralization [37, 38]. An increasing body of evidence indicates that Aβ oligomers play a key role in neuronal dysfunction and development of AD [39–41], whereas Aβ monomer and APP are involved in the development and plasticity of the nervous system and contribute to cognitive performance and memory [42]. A vaccine or antibody that specifically targets Aβ oligomers but not Aβ monomer or APP may have better therapeutic potential [39]. Recently, an AβO monoclonal antibody produced by Biogen, aducanumab, reduced Aβ levels in the brain and slowed the clinical recognition decline of patients with AD [4], implying that the specificity of antibodies for Aβ oligomers is important for the development of AD therapeutics.

To imitate the epitope of Aβ oligomers, ABri and 3A vaccines were developed. ABri is a peptide that has no sequence homology to Aβ or other human proteins and induces British amyloidosis. Its polymerized forms may simulate the conformational structures of Aβ oligomers and induce antibodies against Aβ oligomers [43]. 3A, a peptide with a nonhuman random sequence, may aggregate to oligomers that mimic Aβ oligomers and elicit antibodies recognizing Aβ oligomers [44]. Like Aβ, ABri and 3A themselves are not epitopes of Aβ oligomers and may form oligomeric mimotopes only after aggregation. However, the titer of antioligomer antibodies induced by these two vaccines did not easily reach the desired levels, because the immunogenicity of the formed mimotopes could not be readily improved by carriers. AD02 was another vaccine targeting Aβ aggregates. It was composed of keyhole limpet hemocyanin (KLH) and an epitope peptide simulating the conformation of the Aβ_1–6_ fragment. However, AD02-induced antibodies specifically recognized Aβ fibrils rather than Aβ oligomers, which may limit its therapeutic potential [39].

Aβ oligomers generally exist in varied forms with a wide range of molecular weights and are unstable in solution [45, 46], which brings enormous difficulties to obtaining the oligomer-specific epitopes. In the present study, we first obtained a series of peptides that simulated the specific epitopes of Aβ oligomers by screening the random 12-peptide libraries using IVIG-AβO as target proteins. Twenty peptides were isolated, and eight were expressed on the surface of EBY100 *S. cerevisiae* and immunized C57BL/6 mice. All eight vaccines elicited antibodies against the epitope peptides, but only one candidate, AOE1 with L2 peptide as a mimotope, elicited oligomer-specific antibodies. Moreover, when expressed on hepatitis B core-based virus-like particles or chemically conjugated to KLH, L2 still did not induce antibodies against Aβ oligomer, suggesting that both L2 and yeast EBY100 contributed the formation of simulated conformation of the mimotope. These results demonstrated that although the eight synthetic epitope peptides bound to IVIG-AβO in vitro and the induced antibodies bound to mimotopes themselves at high titers, the most expressed epitope on the carrier did not simulate the conformation of the epitope peptides. Therefore, it is very difficult for the epitope peptides to simulate a spatial three-dimensional conformation of oligomer, which may be a main barrier for the development of amyloid conformation-specific vaccine. Fortunately, we happened to obtain AOE1, which induced generation of antibodies against Aβ oligomer. In a strict way, AOE1 vaccine is the first vaccine prepared by the mimotope of Aβ oligomers. AOE1 attenuated the cognitive dysfunction of APP/PS1 transgenic mice by reducing Aβ burden, especially Aβ oligomers with the molecular size of 40–60 kDa, including a 56 kDa soluble Aβ oligomer (Aβ*56) that impaired long-lasting synaptic plasticity and disrupted cognition independently of neuron loss or plaque deposition in AD [47]. Although the titers of anti-Aβ oligomer antibodies induced by AOE1 were not very high, AOE1 immunization still exhibited a remarkably therapeutic effect, which may be due to the behavioral improvement of AD mice being closely linked to the reduction of Aβ oligomers rather than amyloid plaques or overall Aβ levels [48].

Neuroinflammation induced by microglia and astrocytes is associated with the pathogenesis of AD [49, 50]. Aβ oligomers are critical initial factors in activating the microglia and astrocytes to release inflammatory factors and amplify neurodegeneration [51, 52]. In the present study, AOE1 vaccine significantly decreased the activation of astrocytes and microglia in the hippocampus and cortex of AD transgenic mice and may have partly contributed to the attenuation of AD pathologies and cognitive deficits. The side effect induced by Aβ-specific T-cell reactions has been one obstacle in the development of AD therapeutic vaccines. In this work, AOE1 did not activate Aβ-specific T cells or induce microhemorrhage, discarding the disadvantages of previous Aβ vaccines. Like other vaccines with yeast as a carrier, AOE1 was characterized by easy preparation, good safety, and low cost [53, 54], suggesting that AOE1 holds potential promise for AD treatment.

## Conclusions

We generated a novel vaccine, AOE1, with an Aβ oligomer-specific mimotope that could induce antibodies with high specificity to Aβ oligomers. AOE1 immunization attenuated Aβ pathologies and cognitive deficits in transgenic mice with AD, decreased the overactivation of glial cells, and did not induce microhemorrhage in the brains of AD mice. These findings suggest that AOE1 may be a safer and more effective vaccine for the treatment of AD than the ones developed to date.

## Abbreviations

Aβ: β-Amyloid peptide
AβO: β-Amyloid peptide oligomer-specific antibodies
AD: Alzheimer’s disease
APP: Amyloid precursor protein
DMSO: Dimethyl sulfoxide
DTT: Dithiothreitol
ECL: Enhanced chemiluminescence
ELISA: Enzyme-linked immunosorbent assay
ELISPOT: Enzyme-linked immunospot
FITC: Fluorescein isothiocyanate
GFAP: Glial fibrillary acidic protein
HFIP: Hexafluoroisopropanol
Iba-1: Ionized calcium-binding adaptor molecule-1
IFN-γ: Interferon-γ
IgG: Immunoglobulin G
IHC: Immunohistochemistry
IL-4: Interleukin 4
IOD: Integrated optical density
ITI: Intertrial interval
IVIG: Intravenous immunoglobulin G
KLH: Keyhole limpet hemocyanin
NC: Nitrocellulose
NOR: Novel object recognition
OD: Optical density
PBST: PBS with Tween-20
PMA: Phorbol 12-myristate 13-acetate
RIPA: Radioimmunoprecipitation assay
RT: Room temperature
ThS: Thioflavin S
WT: Wild type
